# Function, Structure, and Transport Aspects of ZIP and ZnT Zinc Transporters in Immune Cells

**DOI:** 10.1155/2018/9365747

**Published:** 2018-10-02

**Authors:** Bum-Ho Bin, Juyeon Seo, Sung Tae Kim

**Affiliations:** ^1^Department of Molecular Science and Technology, College of Natural Sciences, Ajou University, Suwon 16499, Republic of Korea; ^2^Division of Pathology, Department of Oral Diagnostic Sciences, School of Dentistry, Showa University, Tokyo 142-8555, Japan; ^3^Basic Research & Innovation Division, AmorePacific R&D Unit, Yongin 17014, Republic of Korea; ^4^Department of Pharmaceutical Engineering, Inje University, Gimhae 50834, Republic of Korea

## Abstract

Zinc is an important trace metal in immune systems, and zinc transporters are involved in many immune responses. Recent advances have revealed the structural and biochemical bases for zinc transport across the cell membrane, with clinical implications for the regulation of zinc homeostasis in immune cells like dendritic cells, T cells, B cells, and mast cells. In this review, we discuss the function, structure, and transport aspects of two major mammalian zinc transporter types, importers and exporters. First, Zrt-/Irt-like proteins (ZIPs) mediate the zinc influx from the extracellular or luminal side into the cytoplasm. There are 14 ZIP family members in humans. They form a homo- or heterodimer with 8 transmembrane domains and extra-/intracellular domains of various lengths. Several ZIP members show specific extracellular domains composed of two subdomains, a helix-rich domain and proline-alanine-leucine (PAL) motif-containing domain. Second, ZnT (zinc transporter) was initially identified in early studies of zinc biology; it mediates zinc efflux as a counterpart of ZIPs in zinc homeostasis. Ten family members have been identified. They show a unique architecture characterized by a Y-shaped conformation and a large cytoplasmic domain. A precise, comprehensive understanding of the structures and transport mechanisms of ZIP and ZnT in combination with mice experiments would provide promising drug targets as well as a basis for identifying other transporters with therapeutic potential.

## 1. The Role of Zinc Transporters in Immune Cells

Zinc plays critical roles in the immune system, and its transport proteins, called zinc transporters, are reportedly involved in many immune responses [1, 2]. Two types of zinc transporters are conserved in mammals: Zrt-/Irt-like protein (ZIP) zinc transporters, which increase cytoplasmic zinc level by zinc import, and ZnTs (zinc transporters), which decrease cytoplasmic zinc level by zinc export [1].

ZIP6 and ZIP10 were initially reported as the key mammalian zinc transporters in the regulation of immune cell function [3]. Dendritic cells (DCs) that are differentiated from hematopoietic bone marrow progenitor cells play important roles in presenting antigens to T cells as messengers between the innate and the adaptive immune systems [4]. When DCs are exposed to lipopolysaccharides (LPS), a ligand for toll-like receptor 4 (TLR4) is fragmented from the outer membrane of gram-negative bacteria, and intracellular zinc level is drastically decreased for maturation [3]. LPS stimulation activates the TLR4-TRIF axis, resulting in downregulation of zinc importers ZIP6 and ZIP10 and upregulation of zinc exporters Znt1 and Znt6. Similar to LPS, the zinc-chelating reagent, TPEN (*N*,*N*,*N*′,*N*′-tetrakis (2-pyridylmethyl)-ethylenediamine), induces a significant upregulation of the surface expression of the major histocompatibility complex (MHC) class II and CD86, leading to DC maturation. ZIP6 is also an important molecule in CD4 T cells [5]. CD4 T cells are white blood cells that mature in the thymus from thymocytes and play a central role in humoral immunity [6]. CD4 T cells express the T cell receptor (TCR) on their surface, which recognizes antigenic peptides bound to MHC-*α* in DCs or other antigen presenting cells. TCR activation induces naïve CD4 T cells to differentiate into several T cell subsets including Th1, Th2, Th17, and Treg cells. When TCR signaling is activated by DCs, the ZIP6-mediated zinc level at spatially restricted regions near the immunological synapse between T cell and DC is increased [5]. This zinc accumulation inhibits tyrosine phosphatase SHP-1 recruitment for the negative regulation of TCR signaling, leading to the activation of the TCR-LCK-ZAP70 pathway for cell proliferation and cytokine production. TCR activation induces ZIP8 expression in human T cells [7]. ZIP8 is localized to the lysosomal membrane to transport zinc from the luminal side to the cytoplasm of T cells during TCR activation. An increased ZIP8-mediated zinc level blocks calcineurin activity, thereby mediating the phosphorylation of CREB for IFN-gamma transcription. This result supports several well-known reports that zinc deficiency reduces the production of cytokines such as IL-1, IL-2, and IFN-*γ* [7–10].

Recent advances have demonstrated that ZIP8 is important in various immune cells associated with innate immune function [11]. NF-*κ*B, a central transcription factor that regulates innate immune responses including proinflammation pathways, regulates ZIP8 expression in monocytes, macrophages, DCs, and nonprofessional cells such as lung epithelia. When TNF-*α* and LPS bind to their receptors, I*κ*B kinase (IKK) is activated, leading to I*κ*B*α* protein phosphorylation. Phosphorylated I*κ*B*α* is then dissociated from NF-*κ*B, which activates NF-*κ*B to translocate to the nucleus for the transcription of cytokines and ZIP8. ZIP8-mediated zinc upregulation inhibits IKK upon binding to a specific site within the kinase domain. Thus, ZIP8 negatively regulates the NF-*κ*B pathway, indicating that the zinc-ZIP8-NF-*κ*B axis plays crucial roles during host defense.

B cells play central roles in the adaptive immune system by producing antibodies. Though ZIP10 is enriched in the epidermis for epidermal progenitor survival [12, 13], ZIP10 is also essential for B cell survival and proper functioning [14, 15]. ZIP10-depletion during early B cell development induces a significant reduction in the B cell population by increasing apoptosis [15]. In the normal state, ZIP10-mediated intracellular zinc efficiently inhibits caspases. However, when ZIP10 is depleted, intracellular zinc level is decreased, leading to the activation of caspases for apoptosis. ZIP10 is also important for B cell antigen receptor (BCR) signaling [14]. ZIP10-depleted mature B cells proliferated poorly after BCR cross-linking due to the dysregulation of BCR signaling. In these cells, CD45R phosphatase activity for BCR signaling is reduced. Taken together, ZIP10 is indispensable for immature and mature B cell maintenance for humoral immune responses. Interestingly, in the chicken DT40 cells, ZIP9 is crucial for BCR signaling by regulating AKT and ERK [16].

The zinc transporter can indirectly modulate immune responses. Although ZIP4 is also expressed in the human epidermis [17], it is mainly expressed in the brush border of the small intestine for zinc uptake from diet [18]. Thus, mutations in ZIP4 lead to a zinc deficiency in the body. Though ZIP4 is expressed in intestinal enterocytes, mutations in ZIP4 leads to acrodermatitis enteropathica (AE), a type of skin disease involving blistering of the skin and enhancement of bacterial infection. In AE patients and mice with zinc-deficient diets (ZD), the skin pathogenic mechanism is associated with Langerhans cells (LCs) [19]. Zinc deficiency leads to an impaired production of TGF-*β*1, which is essential for LCs, leading to, at least in part, LC loss. Zinc deficiency itself could increase the apoptosis rate, further leading to LC loss in the skin of AE patients and ZD mice. The loss of LCs increases the accumulation of adenosine triphosphate (ATP), released from damaged keratinocytes, in the epidermis, leading to irritant contact dermatitis. Similarly, zinc transporters restricted to normal human cells, except immune cells, can mediate immune responses since normal human cells release many cytokines and compounds that can stimulate immune cells.

Many studies have highlighted the importance of the ZIP members in immune response; however, relatively little information about the ZnT members is available. ZnT5-mediated zinc homeostasis in allergic response is a well-known phenomenon. ZnT5-depleted mast cells show reduced Fc epsilon receptor I- (Fc*ϵ*RI-) induced cytokine production [20]. ZnT5 regulates Fc*ϵ*RI-induced translocation of protein kinase C (PKC) to the cell membrane for NF-*κ*B-dependent cytokine production. In addition, ZnT8, which is expressed in *β* cells and associated with diabetes [21, 22], may modulate immune cells since diabetes disturbs the body's immune system.

Taken together, zinc transporters play indispensable roles in immune cells. Therefore, understanding the structure and transport mechanism of zinc transporters could offer new approaches to develop drugs to treat immune dysfunction-related diseases.

## 2. Structure of ZIPs

### 2.1. ZIP Family

**Figure 1:**
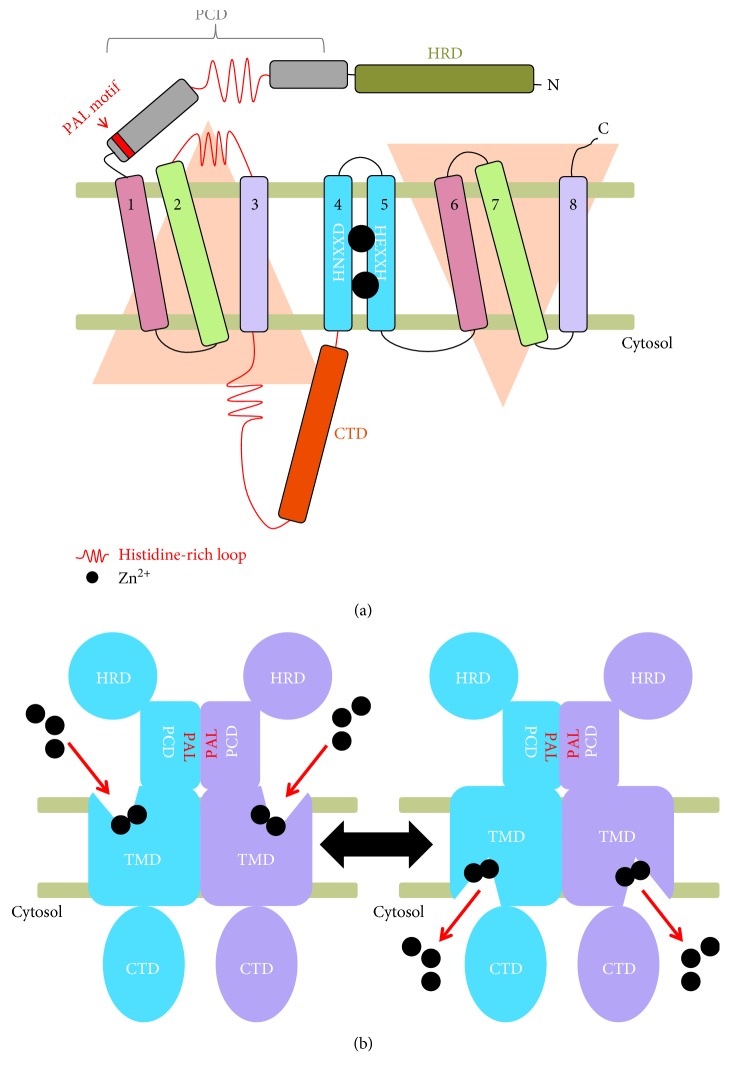
Overview of ZIP. (a) Topological model of ZIP. ZIP is composed of 8 TMDs with a large N-terminal domain including a PCD and HRD as well as a CTD. The pseudosymmetric TMs are shown in the same color. Zn^2+^ binds to the active site made up of TMD4 and 5 via the conserved HNXXD and HEXXH motifs. (b) Transport mechanism. ZIP has two major conformations, the inward-open and the outward-open conformations. The PAL motif and TMDs are involved in dimerization.

Based on sequence analyses, ZIP family members contain 8 transmembrane domains (TMDs) as membrane transport proteins, with a cytoplasmic region between TM3 and TM4 [23, 24] (Figure 1(a)). The ZIP family is divided into four subfamilies according to sequence similarity: subfamilies I, II, gufA, and LIV-1. Subfamilies I, II, and gufA are phylogenetically closely related [24]. The LIV-1 subfamily is associated with the estrogen-regulated gene *LIV-1* and is mainly found in mammals [24]. Only LIV-1 subfamily members contain the HEXXH motif within TM5; they also exhibit diverse lengths of the N-terminal extracellular domain (ECD) [24] (Figure 1(a)). The ECD is very small or is not present in subfamilies I, II, and gufA, but is highly variable in LIV-1 [1, 25]. In mammals, 14 ZIP family members have been identified and are referred to as ZIP1–14. ZIP1, 2, and 3 are classified as the ZIP II subfamily. ZIP9 belongs to the ZIP I subfamily, and ZIP11 to the gufA subfamily. The remaining 9 ZIPs are classified as LIV-1 subfamily members, and the complicated roles of these ZIPs in mammalian cells may be associated with their ECDs. The largest cytoplasmic domain (CTD) of ZIP (ZIP-CTD) is located between TM3 and TM4. ZIP4-CTD is ubiquitinated in response to metal cytotoxicity [26]. However, the roles of ZIP-CTD are unknown [23].

### 2.2. Structural Overview

Among 14 mammalian ZIP family members, only ZIP13, a LIV-1 subfamily member, has been successfully purified from an insect overexpression system owing to bottlenecks in the overexpression and purification procedures for other mammalian ZIPs [23]. For precise biochemical and structural analyses, prokaryotic ZIPs have been applied to mammalian ZIP research. ZIPB from *Bordetella bronchiseptica* was overexpressed, purified from *Escherichia coli*, and crystallized to determine the X-ray crystal structure [27]. The crystal structure of ZIPB revealed a novel 3 + 2 + 3-TM architecture; the first 3 TMs (TM1 to TM3) can be superimposed on the last three TMs (TM6–8) by rotating 180°, and TM4 and TM5 are symmetrically related and sandwiched by the two 3-TM repeats. This feature is unique among transporters.

### 2.3. Zinc Binding Site

The crystal structure of ZIPB demonstrated an inward-open conformation with a binuclear zinc center in the transport pathway, suggesting the existence of an outward-open conformation [27] (Figure 1(b)). The binuclear zinc center is formed by TM4 and TM5 with several conserved polar amino acid residues (Figure 1(a)). The HEXXHE motif, a LIV-1-specific motif, is found in TM5, and a comparative computational model of the ZIPB structure indicated that this motif serves as the binuclear zinc center. The ZIPB structure shows multiple conserved zinc binding sites near the metal exit cavity, indicating that these sites constitute a route of zinc release to the cytoplasm. Therefore, it is possible that the conserved histidine-rich motif found in mammalian ZIPs near TMs includes multiple zinc binding sites to transfer zinc efficiently from the binuclear zinc center.

### 2.4. Extracellular Domain

The crystal structure of only the ECD of ZIP4 (ZIP4-ECD) from *Pteropus alecto* has been revealed [25]. The ZIP4-ECD is stabilized by 4 conserved disulfide bonds. The ZIP4-ECD has two structurally independent subdomains with 14 *α*-helices, a helix-rich domain (HRD), and a PAL motif-containing domain (PCD) that is observed only in LIV-1 subfamily members. In particular, the HRD is conserved in ZIP4 and ZIP12 among 9 LIV-1 subfamily members, and others lack this domain. The HRD forms a novel protein fold from 156 amino acids from the N-terminus of ZIP4 and is composed of 9 *α*-helices, indicating a high *α*-helical content (73%) (Figure 1(a)). The PCD is conserved in ZIP4, 5, 6, 8, 10, and 12, but is lacking in ZIP7 and ZIP13. Interestingly, ZIP7 and ZIP13 are localized to intracellular compartments, like the ER and Golgi apparatus [28, 29], suggesting that the HRD and PCD are associated with their localization. The PCD, a C-terminal subdomain of ZIP4-ECD, forms from 130 amino acids; it is composed of 5 *α*-helices and exhibits a pair of helix-turn-helix folds. The longest *α*-helix of PCD possesses the PAL motif, which is structurally important for dimerization and stabilization. All of the helices in the PCD are involved in dimerization, and the PAL motif is at the center of the dimerization interface. However, ZIP13, which contains a short ECD with a degenerated PAL motif, also forms a dimer, suggesting that PCD may not be crucial for dimerization in ZIP13. PCD also contains an unstructured histidine-rich loop with potential roles in metal sensing, transport, or storage. According to a recent proposal, the cellular prion protein (PrP^C^) evolved from ZIP approximately 500 million years ago, since the cysteine-flanked core (CFC) domain within PCD shows high structural similarity to PrP^C^ [30]. The PAL motif in PrP also represents a dimerization interface, similar to ZIP4-ECD [27, 30].

### 2.5. Oligomerization

ZIP13 has been purified as a homodimer [23] (Figure 1(b)). A purified prokaryotic ZIP, ZIPB in *n*-dodecyl-*β*-d-maltopyranoside (DDM) ran as a dimer on size exclusion columns based on their apparent detergent-solubilized molecular weights [27]. Interestingly, purified ZIPB shows two species, consistent with monomeric and dimeric states, and both species crystallized under identical conditions [27]. Moreover, purified ZIP13 sometimes ran as a monomer in blue native- (BN-) PAGE and Western blot analyses [23]. The ZIP monomer itself forms a perfect metal transport channel [27] and accordingly may act as a metal transporter. ZIP6 and ZIP10 reportedly form a heterodimer that is critical for the epithelial-to-mesenchymal transition [31]. Thus, the dimeric state might have further functions, for example, in signal transduction.

### 2.6. Transport Mechanism

The zinc transport mechanism is still controversial and depends on the type of ZIP. Early cell-based assays using isotopes have revealed that zinc flux via ZIP2 is time-, temperature-, and concentration-dependent and saturable with an apparent *K*_*m*_ of 3 *μ*m in transfected K562 erythroleukemia cells [32]. ZIP2 activity was not affected by ATP, K^+^, or Na^+^ gradients, but was significantly stimulated by HCO_3_^−^ treatment with high affinity to the first zinc and the next cadmium, suggesting a Zn^2+^-HCO_3_^−^ symporter mechanism [32]. ZIP8, which is involved in cadmium-induced toxicity in the testis [33], shows a Mn^2+^-HCO_3_^−^ symporter mechanism in ZIP8 cDNA-stable retroviral-infected mouse fetal fibroblasts in physiological conditions [34]. Previous reports have shown that the *K*_*m*_ for Cd^2+^ uptake by ZIP8 is 0.62 *μ*M, and the *K*_*m*_ for Mn^2+^ uptake is 2.2 *μ*M with a low affinity to Zn^2+^. However, ZIP8 shows a Zn^2+^-HCO_3_^−^ symporter mechanism with an influx of two HCO_3_^−^ anions per one Cd^2+^ (or one Zn^2+^) cation, that is, electroneutral complexes, in an electrogenicity study of ZIP8 cRNA-injected *Xenopus* oocytes [35]. Cadmium (Cd^2+^) and zinc (Zn^2+^) uptake have *V*_max_ values of 1.8 ± 0.08 and 1.0 ± 0.08 pmol/oocyte/h, and *K*_*m*_ values of 0.48 ± 0.08 and 0.26 ± 0.09 *μ*M. Many other metal transporters on the surface of mammalian cells might obscure these effects. *Xenopus* oocytes have negligible amounts of interfering transporters, but proper protein folding and distribution are sometimes a bottleneck in the determination of the precise transport mechanism. To resolve this issue, a proteoliposome-based kinetic analysis using purified ZIPB, a prokaryotic ZIP, has been applied to determine the transport mechanism. Similar to a zinc-selective channel, zinc flux via ZIPB was nonsaturable for a zinc concentration of up to 2 mM and was electrogenic [36], and this transport mechanism is consistent with ZIP2 [37]. However, this is inconsistent with the results of previous cell-based and electrogenic analyses, further emphasizing the lack of clarity regarding the zinc transport mechanism of mammalian ZIPs.

The crystal structure of ZIPB revealed that the eight TMs are grouped into two units, one including TM1, 4, 5, and 6 and another including the other four TMs as a traditional membrane carrier involving significant conformational changes [27] (Figure 1(b)). The solved crystal structure indicating an inward-open conformation of ZIPB without open entrances toward the extracellular space has a filter-like histidine 177 (H177) in the metal pathway below the metal binding sites toward the cytosol. H177 adopts two conformations for metal release from metal binding site 1. Below H177 toward the cytosol, where the exit cavity is located, a histidine-rich loop connecting TM3 and 4 probably facilitates zinc release as a “metal sink.” Though a proteoliposome-based kinetic analysis has revealed that ZIPB mediates zinc influx as a channel, the crystal structure of ZIPB implies that ZIPB may act as a transporter [27, 36]. The crystal structure for the outward-open conformation is needed to resolve this inconsistency. Zinc influx through ZIPB is extremely slow compared with typical ion channels; accordingly, ZIPB might adopt a unique mechanism that it uses to intermediate between typical transporters and channels.

## 3. Structure of ZnTs

### 3.1. ZnT Family

**Figure 2:**
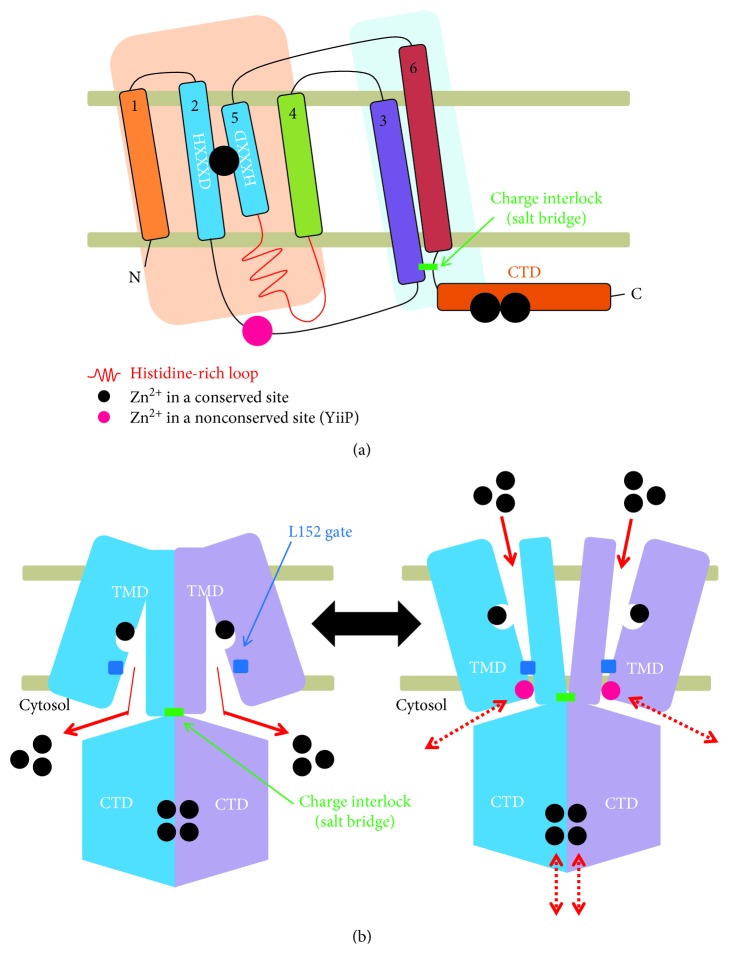
Overview of ZnT. (a) Topological model of ZnT. TMDs of ZnT are divided into two groups; TM1, 2, 4, and 5 form one bundle and TM3 and 6 form the other bundle. TM2 and TM4 share the Zn^2+^ binding site via HXXXD motifs. TM3 and 6 trigger the charge interlock that stabilizes the dimeric conformation. (b) Transport mechanism. ZnT has two major conformations, the inward-open and outward-open conformations, similar to ZIP. CTDs contain 4 Zn^2+^ that are not transported across the lipid bilayer but contribute to the stabilization of dimerization in a zinc-dependent manner. In the YiiP crystal structure, one Zn^2+^ is found in the link between the TMD and CTD (pink circle).

ZnTs belong to a superfamily of cation diffusion facilitators (CDF) observed in a wide range of taxa, including plants, bacteria, and fungi [38]. In mammals, at least 10 ZnT members have been identified [1, 38]. They are predicted to have six TMDs, except for ZnT5, which has additional TMDs, and their N- and C-termini face the cytoplasm, unlike those of ZIPs [1, 38] (Figure 2(a)). ZnTs show a histidine/serine-rich loop with diverse lengths between TM4 and TM5. Interestingly, ZnTs contain a large CTD at the C-terminus with a copper chaperon-like architecture, despite a lack of sequence homology [39]. This domain is important in diabetes research since variants of ZnT8 in the CTD are associated with diabetes risk [40]. Especially, variant W325R in the CTD increases the risk of developing diabetes by affecting thermostability. Moreover, ZnT8 is a major autoantigen in type 1 diabetes, implying that the variant conformation of the CTD affects antigen recognition [41].

Though the first ZnTs were identified by selecting zinc-resistant baby hamster kidney cells [42], the overexpression and purification of mammalian ZnTs such as ZnT8 have become a success in recent years [43]. Instead, a prokaryotic CDF member has been well studied. YiiP from *E. coli* has been overexpressed, purified [44], and finally crystallized to determine its atomic structure [39].

### 3.2. Structural Overview

The crystal structure revealed that YiiP exhibits a unique Y-shaped structure formed by two monomers [39] (Figure 2(b)). Two TMDs of each monomer swing outward and plunge into the membrane at a 60° angle. The void space between two TMDs from each monomer is thought to be filled with phospholipids *in vivo*. In each monomer, helixes in the TMD could be divided into two groups; TM1, 2, 4, and 5 form a compact four-helix bundle and TM3 and 6 cross over in an antiparallel conformation outside the bundle to support the dimerization between monomers by conserved hydrophobic interactions at each TMD-TMD interface [39, 45]. At the dimer interface, Lys77 in TM3 and Asp207 from the intracellular loop that connects TM6 and the CTD stabilize the TM3-TM6 pairs by forming four interlocked salt bridges.

The crystal structure of YiiP reveals that the helix-breaking proline residue is lacking in TMDs of YiiP, indicating the limited conformational flexibility when zinc is transported [39]. However, TM5 is relatively short, with 2 residues among 4 coordinating residues in a tetrahedral arrangement for binding to one Zn^2+^ [45], suggesting that TM5 movement is a major event in zinc transport. The remaining 2 residues for completing the tetrahedral coordination for single Zn^2+^ binding are located on TM2. These evolutionarily conserved Zn^2+^ binding sites are located near the bottom of the extracellular cavity in the structure of YiiP, indicating that the solved crystal structure is the outward-open conformation (Figure 2(b)). Though the intracellular cavity is between the TMD and CTD, Zn^2+^ binding residues are not present, further suggesting that the solved crystal structure of YiiP is not the inward-open conformation.

Recent cryoelectron microscopic analysis using a YiiP homolog from *Shewanella oneidensis* shows the inward-open conformation for the cytoplasmic cavity [46]. The cryoelectron structure reveals that YiiP forms a still monodimer, without apparent conformational changes in CTDs. However, the cytoplasmic sides of TMDs involve significant conformational changes, producing dimeric interactions via TM3 and 6, resulting in interactions between the monomers in the whole regions of TM3 and TM6. Though the periplasm region of the compact four-helix bundle (TM1, 2, 4, and 5) moves closer to the two-helix bundle (TM3 and TM6) in the cryoelectron structure, the cytosol part of the four-helix bundle moves away from the two-helix bundle. Therefore, the cryoelectron structure of YiiP has a cytoplasmic cavity with an inward-open conformation (Figure 2(b)).

### 3.3. Zinc Binding Site and Oligomerization

Each YiiP monomer in the crystal structure has three Zn^2+^ binding sites [39, 45] (Figure 2). For transport across the lipid bilayer, Zn^2+^ is located in the center of the TMD by tetrahedral coordination via four residues from Asp45, Asp49 in TM2 and His153, and Asp157 in TM5. This coordination indicates that Zn^2+^ is completely surrounded by amino acid residues from TMs, and not by any other outer-shell constraints, like a water molecule. Thus, no breaking of the outer shell is needed to mediate Zn^2+^ release and accordingly little reorientation of TM2-TM5 could efficiently induce Zn^2+^ binding or release. The tetrahedral coordination prefers Zn^2+^ and Cd^2+^, not Fe^2+^, Ca^2+^, Mn^2+^, and so on, further suggesting that YiiP is a zinc transporter.

The intracellular loop (IL1) of YiiP that connects TM2 and TM3 includes a single Zn^2+^ binding site [39, 45] (Figure 2(a)). These sites are only observed in the YiiP sequences, indicating that they may not be conventionally important in ZnTs. However, several observations suggest that they may be functionally significant in some ZnT family members: (1) His71 that serves as a Zn^2+^ binding residue interacts with Gln203 in TM6 of the other monomer, (2) IL1 contains many zinc binding residues in some ZnT family members, and (3) this Zn^2+^ binding site is located near a cluster of highly conserved residues in the cytosolic region of TM3 at the dimer interface. Therefore, Zn^2+^ binding may be important for dimerization via direct or indirect interactions (hydrogen bonds or van der Waals forces) across the dimer interface.

Four bound Zn^2+^ are found at the CTD interface in the YiiP crystal structure [39, 45] (Figure 2(b)). These Zn^2+^ are coordinated by highly conserved residues, including extensive outer-shell constraints and water molecules. Some of the neighboring residues interact with these conserved residues, resulting in bidentate hydrogen bonds, thereby establishing a well-arranged network of outer-shell interactions. In addition, the bound Zn^2+^ shows resistance to EDTA chelation. As a result, Zn^2+^ binding at the CTD interface stabilizes the CTD-CTD interaction, thus strengthening the dimerization of YiiP monomers.

### 3.4. Cytoplasmic Domain and Oligomerization

The CTD exhibits a high structural similarity with that of the copper chaperone Hah1 [47, 48]. Despite low sequence similarity between the CTD and Hah1, they share an *αβ*-*βα* core structure. Since metallochaperones and their targets share a metallochaperone domain, a zinc chaperone that binds to YiiP and transfers zinc might exist. However, to date, a zinc chaperone has not been reported.

In the crystal structure of YiiP, the two positively charged protein surfaces of the CTD interface are held by four Zn^2+^ ions [39, 45]. If Zn^2+^ is absent from these sites, electrostatic repulsion pushes each CTD monomer away. The site-directed fluorescent resonance energy transfer (FRET) measurements using the CTD-labeled full-length purified YiiP protein shows that Zn^2+^ binding triggers hinge movement, pivoting around the (Lys77-Asp207)_2_ charge interlock on the cytoplasmic membrane surface. However, the cryoelectron structure of the apo-zinc form of a YiiP homolog based on 2D crystallization is identical to the crystal structure of the zinc-bound form of a CTD, without any apparent conformational changes [46]. These results imply that CTDs are important for the stabilization of the dimeric state, and thus TMD movement for zinc transport may occur without substantial CTD conformational changes.

The crystal structures of several bacterial CTD homologs have been solved and used for modeling analyses of mammalian ZnT [49, 50]. The apo and zinc forms of the CTD in CzrB from *Thermus thermophilus* reveal that only CTDs without TMDs could form a V-shaped dimer, and they show substantial zinc-dependent conformational changes [49]. Three Zn^2+^ ions are found in the monomer-monomer interface and their binding triggers a dramatic reorientation of the two monomers by a shift of about 40° through electrostatic repulsion. The crystal structure of the CTD from another CDF family member, MamM, in *Magnetospirillum gryphiswaldense* has also been solved [50]. However, only the apo form of the CTD has been solved, since the cation-bound form is limited by severe precipitation. The MamM-CTD also forms a V-shaped dimer, and metal binding triggers conformational changes. In general, CTD structures without TMDs are distinct from the full-length structure of YiiP. Therefore, the action/role of the CTD is still controversial. In the full-length structure of YiiP, CTDs are fastened by the charge interlock, which may prevent the dramatic movement of the CTD, as observed in the CTD-only structures of CzrB and MamM [39, 45, 49, 50].

### 3.5. Transport Mechanism

Using the purified YiiP protein, zinc efflux occurs via a Zn^2+^/H^+^ antiporter [44]. YiiP mediates Zn^2+^ efflux to the outside of cells or to the luminal side of the intracellular compartment. At the same time, H^+^ ions are transported into the cytoplasm. Therefore, YiiP requires a proton motive force to mediate Zn^2+^ efflux. Indeed, isothermal titration calorimetric analyses have revealed that YiiP-mediated efflux is coupled with proton influx in a 1 : 1 zinc-for-proton exchange stoichiometry [44]. Zn^2+^ binding drives an all-or-nothing change in water accessibility to the transport site by the L152 gate in TM5 [51] (Figure 2(b)).

## 4. Conclusions

ZIPs and ZnTs play pivotal roles in the maintenance of cellular zinc homeostasis. Interestingly, both form dimers and share a traditional transport mechanism, including two major conformational states, that is, inward-open and outward-open. Their zinc binding sites are solved based on the crystal structures, providing insight into the zinc transport mechanism and providing a basis for drug development. Drugs that work as agonists are largely divided into two groups, “potentiators” and “correctors” [52]. Potentiators are compounds that stabilize transporters by binding to the specific site of transporters. Structure and biochemical data indicate that CTDs of ZnTs are involved in protein stability. Based on this, compounds that bind to CTDs of ZnTs can be classified as potentiators. Furthermore, drugs that directly bind near the active pore of transporters to stabilize the active conformation could increase the transport activity and may thus be classified as potentiators. In addition, compounds that regulate the degradation of transporters could be correctors. Many molecules involved in the degradation of zinc transporters are largely unknown. Thus, studies on the degradation and regulatory machinery for zinc transporter maintenance are strongly needed. Compounds that destabilize transporters could be useful as antagonists since the hyperactivation of zinc transporters may be related to immune dysfunction. Furthermore, ECDs of ZIP are very unique, but are similar to prions. Prion binding compounds could be screening targets to develop drugs.

Altogether, additional studies of the ECD and CTD may extend our knowledge of the diverse signaling mechanisms and their roles in disease pathogenesis. Therefore, a more precise understanding of the structures of ZIPs and ZnTs would be helpful to regulate immune cells, treat human diseases, and improve health life.
